# SMICA: Semantic and Multi-Granularity Integration with Constrained Adaptation for Fake Image Detection

**DOI:** 10.3390/jimaging12100522

**Published:** 2026-10-20

**Authors:** Zhiyu Lin, Zhongbo Wang, Tianxin Xu, Songyu Ye, Xiong Xu, Chengzong Peng

**Affiliations:** 1 College of Computer Science (College of Software Engineering), Sichuan University, Chengdu 610065, China; zhiyulin@stu.scu.edu.cn (Z.L.); xtx@stu.scu.edu.cn (T.X.); 2 School of Software and Microelectronics, Peking University, Beijing 102600, China; zhongbowang25@stu.pku.edu.cn; 3 Viterbi School of Engineering, University of Southern California, Los Angeles, CA 90089, USA; songyuye0826@gmail.com; 4 The 10th Institute of China Electronics Technology Group Corporation, Chengdu 610036, China; xuxiong_cd@126.com; 5 Industrial Control and Security Center, Dawning Information Industry Co., Ltd. (SUGON), Chengdu 610225, China; 6 School of Cybersecurity (Xin Gu Industrial College), Chengdu University of Information Technology, Chengdu 610225, China

**Keywords:** fake image detection, image forensics, DINOv2, low-rank adaptation, domain generalization, multimedia forensics, multi-granularity feature fusion

## Abstract

The rapid development of generative and image-editing models has increased the prevalence of manipulated and synthetic images, creating substantial risks for security-sensitive applications. Most existing detectors target a single forensic domain and rely on domain-specific priors, limiting transfer to heterogeneous scenarios and unseen forgery types. We propose SMICA, a semantic and multi-granularity integration framework with constrained adaptation for fake image detection (FID). SMICA uses a frozen DINOv2 backbone with Low-Rank Adaptation (LoRA), enabling task-specific feature adaptation while updating only a small fraction of the model parameters. It further fuses the classification (CLS) token, Patch Mean, and Patch Max to capture global semantics, distributed local statistics, and sparse high-response cues. Adaptive Sharpness-Aware Minimization (ASAM) regularizes the trainable parameters toward flatter solutions during training. Under the unified OpenMMSec evaluation protocol, SMICA achieves AUC scores of 0.9608, 0.9685, 0.8809, and 0.8743 on Deepfake, AI-Generated Content (AIGC), Image Manipulation Detection (IMD), and Document Image Forgery Detection (Doc), respectively, yielding a macro-average AUC of 0.9211 while optimizing only 2.50% of the total parameters. These results show that combining multi-granularity forensic evidence with parameter-efficient constrained adaptation provides an effective solution for generalizable FID.

## 1. Introduction

### 1.1. Background

The rapid proliferation of generative AI and image-editing tools has made fake images a widespread security and societal threat, increasing the importance of fake image detection (FID) [1,2]. FID spans four major forensic subdomains [3]: (1) Deepfake Detection for facial manipulations; (2) Image Manipulation Detection (IMD) for regional alterations in natural images; (3) AI-Generated Content (AIGC) Image Detection for fully synthetic images; and (4) Document Image Forgery Detection (Doc) for textual and structural tampering.

Detection performance has improved substantially within individual forensic domains. Deepfake detectors increasingly exploit pretrained vision representations and parameter-efficient adaptation; for example, DFF-Adapter adapts a frozen DINOv2 backbone with lightweight low-rank modules and fine-grained forgery-type supervision [4]. In other domains, Mesorch [5] targets image manipulation, whereas Effort [6] and CO-SPY [7] address AI-generated image detection. Although effective, these methods are primarily optimized for the artifact characteristics and evaluation protocols of their respective domains.

Recent studies have also begun to address the fragmentation of image forensics from a unified perspective. ForensicHub [3] provides a common benchmark and codebase for detection and localization across Deepfake, AIGC, image manipulation, and document forgery. UniShield [8] approaches unified analysis through an adaptive multi-agent system that coordinates expert detectors according to image characteristics. These studies shift the field toward cross-domain forensics but leave open a distinct question: how can a single image-level detector efficiently adapt one shared visual representation to heterogeneous forgery distributions without domain-specific expert routing?

The four domains nevertheless exhibit substantially different forensic evidence. Manipulation traces vary in spatial extent, semantic relevance, frequency characteristics, acquisition pipelines, and whether discriminative cues are globally distributed or locally concentrated [3]. Domain-specific advances therefore do not directly solve shared representation learning for heterogeneous FID, while expert-based unified systems handle heterogeneity through model selection or coordination rather than a single adapted representation [8]. A monolithic detector must instead remain sensitive to complementary forensic cues across these diverse distributions.

### 1.2. Challenges

Against this background, unified FID presents three main challenges.

First, forgery evidence differs substantially across Deepfake, AIGC, IMD, and Doc, and domain-specific priors often transfer poorly across these heterogeneous distributions.

Second, forensic evidence appears at multiple granularities. Global features capture semantic and structural inconsistencies, whereas sparse local artifacts require patch-level cues; relying on either granularity alone can discard complementary information.

Third, adapting a large vision foundation model to heterogeneous FID requires balancing task-specific adaptation, multi-granularity evidence retention, and resistance to domain-specific overfitting. We address these requirements through restricted adaptation, complementary evidence aggregation, and optimization regularization.

DINOv2 supplies a shared self-supervised representation with an image-level CLS token and spatially distributed patch tokens. LoRA restricts task-specific updates to low-rank subspaces, providing parameter-efficient adaptation. Multi-granularity aggregation combines global semantics, distributed local statistics, and sparse high-response cues from the same token space. ASAM then regularizes the trainable LoRA parameters and classification head by discouraging convergence to sharp, distribution-sensitive solutions. Accordingly, LoRA controls how the backbone is adapted, multi-granularity fusion determines which evidence is aggregated, and ASAM regularizes optimization.

We therefore investigate whether combining restricted low-rank adaptation, multi-granularity token aggregation, and sharpness-aware optimization is more effective for unified FID than unrestricted fine-tuning or single-granularity representations.

### 1.3. Contributions

We propose SMICA, a unified FID framework built around restricted adaptation, complementary evidence aggregation, and optimization regularization. Its main contributions are as follows:

1. We develop a parameter-efficient adaptation scheme for unified FID using a frozen DINOv2 backbone with LoRA. Task-specific updates are confined to low-rank parameter subspaces rather than the full pretrained backbone, enabling controlled adaptation to heterogeneous forensic data.
2. We introduce a multi-granularity aggregation strategy for the DINOv2 token representation without additional learnable parameters. The CLS token, Patch Mean, and Patch Max capture image-level context, distributed local statistics, and sparse high-response cues, respectively, allowing evidence at different spatial granularities to contribute to authenticity discrimination.
3. We incorporate ASAM [9] to regularize the trainable LoRA adapters and classification head during heterogeneous joint training. This complements LoRA’s restriction on where adaptation occurs without adding inference-time modules.
4. We evaluate SMICA under the unified OpenMMSec protocol spanning Deepfake, AIGC, IMD, and document forgery. SMICA achieves strong overall AUC and balanced cross-domain performance while training only 2.50% of the model parameters.

## 2. Related Work

Existing image-forensics research can be grouped into five directions: deepfake detection, image manipulation detection, AIGC image detection, document image forgery detection, and unified fake image detection.

### 2.1. Deepfake Detection

Facial manipulations constitute the primary targets in the Deepfake domain, including face swapping, reenactment, editing, and full-face synthesis [1]. Earlier approaches exploited facial and behavioral inconsistencies [10], spatial manipulation boundaries [11], and frequency-domain generation artifacts [12]. More recent methods increasingly emphasize generalization beyond specific manipulation techniques. SAGNet [13], for example, learns semantic-agnostic forgery artifacts to reduce dependence on limited manipulation-specific training data, while multimodal approaches incorporate complementary audio-visual information [14].

Vision foundation models have also motivated parameter-efficient forensic adaptation. DFF-Adapter [4] uses a frozen DINOv2 backbone with lightweight low-rank adapters throughout the Transformer blocks and jointly optimizes authenticity detection and fine-grained forgery-type classification. It demonstrates the potential of DINOv2-based parameter-efficient adaptation, but is designed specifically for face forgeries and manipulation-type supervision. Unified FID instead requires one representation to accommodate heterogeneous artifacts across substantially different forensic domains.

### 2.2. Image Manipulation Detection

The IMD domain focuses on identifying and localizing splicing, copy-move, and object removal at the pixel level [15]. Early deep approaches combined RGB information with noise-sensitive or manipulation-trace representations [15,16]. Recent approaches exploit reconstruction residuals [17], cross-view prompting with the Segment Anything Model (SAM) [18], and multi-task fusion of boundaries, noise, and object consistency [19].

### 2.3. AIGC Image Detection

Distinguishing real images from content synthesized by generative adversarial networks (GANs) and diffusion models is the central objective of AIGC image detection [20,21,22]. Existing methods exploit systematic artifacts associated with CNN-based generation [23] and forensic traces induced by diffusion-based synthesis [24]. Recent approaches combine foundation models with masked spectral learning [25], fast Fourier transform features [26], and intrinsic noise or local variation cues [27].

### 2.4. Document Image Forgery Detection

Document forensics primarily targets localized alterations in structured materials, such as credentials and invoices [28]. These manipulations often occupy only small regions within homogeneous backgrounds [29]. Earlier approaches exploited compression and resampling traces using learned or machine-learning-based forensic representations [30,31]. Recent approaches exploit dual watermarking-based tamper detection [32], multi-scale attention networks [28], and general-purpose multimodal large language models (MLLMs) adapted for interpretable localization [33].

### 2.5. Unified Fake Image Detection

Evaluating forensic domains in isolation limits cross-domain comparison and practical deployment. ForensicHub [3] addresses this fragmentation with a unified benchmark and modular codebase spanning Deepfake, image manipulation, AIGC, and document forgery. Its analysis highlights substantial distributional and representational differences among these scenarios while providing a common protocol for all-domain detection and localization.

Beyond unified benchmarking, UniShield [8] addresses heterogeneous forgery analysis at the system level. Its adaptive multi-agent framework first analyzes image characteristics and then coordinates suitable expert detectors for detection and localization. This design offers flexibility across diverse scenarios, but achieves unification through expert selection and orchestration rather than a single shared detector.

These developments suggest two complementary directions for unified image forensics: coordinating specialized experts or learning a common representation within a monolithic detector. We focus on the latter, where the key questions are how to adapt a pretrained representation under heterogeneous training distributions, which global and local evidence to retain, and how to regularize the trainable parameters during joint optimization.

### 2.6. Foundation-Model Adaptation for Image Forensics

Large-scale pretrained vision models provide transferable visual representations that have increasingly been adopted for forensic detection. CLIP [34], iBOT [35], and DINOv2 [36] provide strong pretrained representations through multimodal or self-supervised learning, while Low-Rank Adaptation (LoRA) [37] offers a parameter-efficient mechanism for introducing task-specific updates without optimizing all backbone parameters. Recent forensic work further demonstrates the relevance of this direction: DFF-Adapter [4] combines DINOv2 with low-rank adaptation and auxiliary forgery-type supervision for face forgery detection.

These studies motivate pretrained foundation representations and restricted adaptation, but do not by themselves resolve heterogeneous evidence aggregation in unified FID. A monolithic detector must adapt the pretrained representation, organize complementary global and local forensic evidence, and regularize a limited set of trainable parameters under multi-domain optimization. SMICA addresses these requirements through LoRA-based restricted adaptation, multi-granularity token aggregation, and ASAM-based optimization regularization, respectively.

## 3. Materials and Methods

SMICA follows three complementary principles for unified FID: restricted adaptation, complementary evidence aggregation, and optimization regularization. DINOv2 [36] provides a shared self-supervised token representation; LoRA [37] confines task-specific updates to low-rank parameter subspaces; multi-granularity fusion organizes global and local evidence from the adapted tokens; and ASAM [9] regularizes the trainable parameters during heterogeneous joint training.

**Figure 1:**
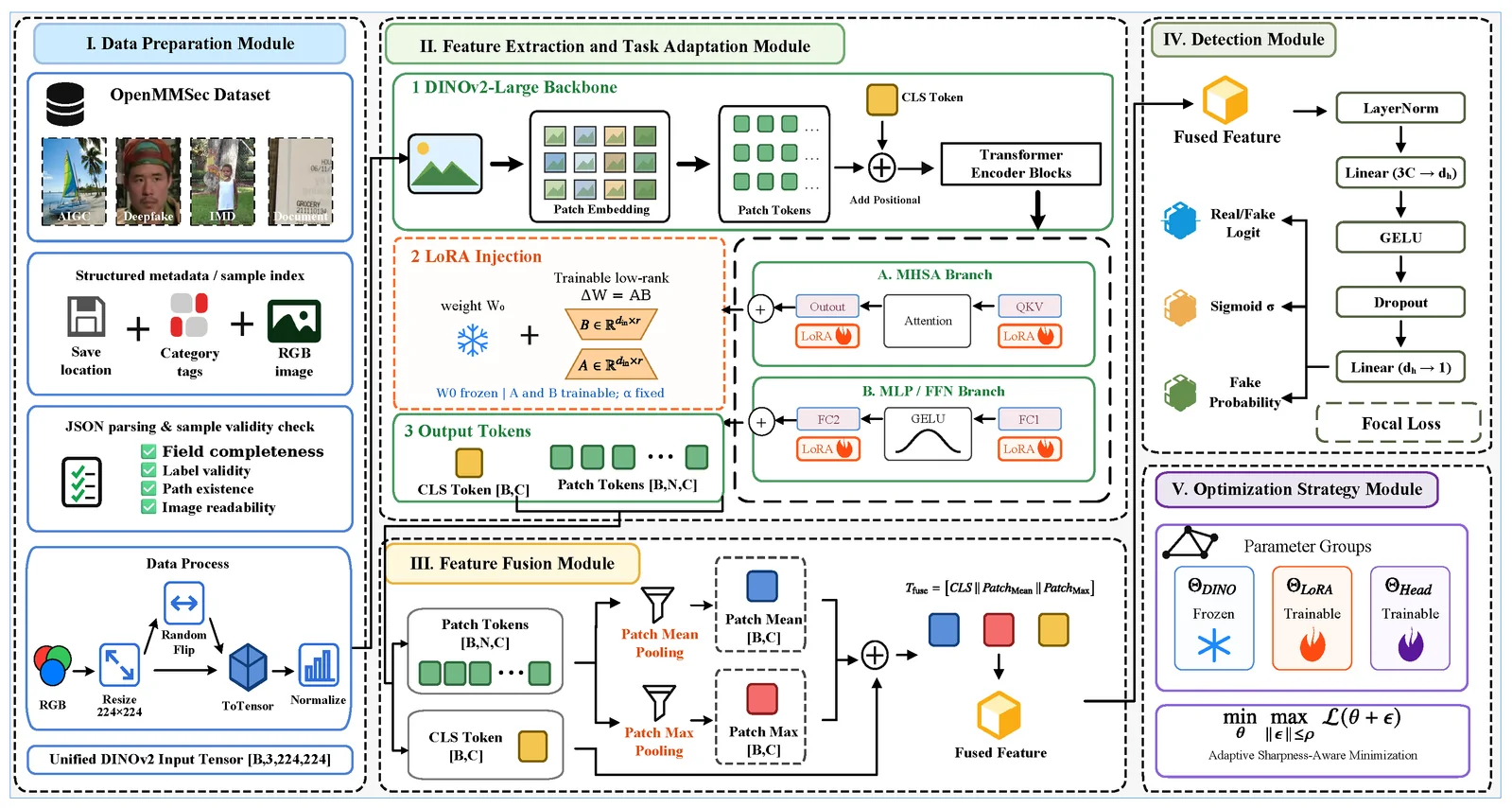
Overview of SMICA. The DINOv2 backbone is frozen, whereas the Low-Rank Adaptation (LoRA) adapters and classification head are optimized. The classification (CLS) token, Patch Mean, and Patch Max representations are concatenated for binary classification.

As shown in Figure 1, SMICA contains five implementation components: the Data Preparation Module, Feature Extraction and Task Adaptation Module, Feature Fusion Module, Detection Module, and Optimization Strategy Module.

The Data Preparation Module standardizes model inputs. The Feature Extraction and Task Adaptation Module uses DINOv2 and LoRA to obtain adapted visual representations. The Feature Fusion Module aggregates token-level evidence across granularities, the Detection Module predicts real/fake probabilities, and the Optimization Strategy Module applies ASAM during training.

### 3.1. Data Preparation Module

Unified FID requires modeling substantially different forensic subdomains. We adopt OpenMMSec (OpenMMSec dataset on the Tianchi platform: https://tianchi.aliyun.com/dataset/210595 (accessed on 9 September 2026).) as the benchmark for Deepfake, AIGC, IMD, and document-forgery scenarios. According to the official release, OpenMMSec contains 333,583 images from 19 public forensic datasets and more than 10 real-image sources. The generalization split uses 26 forgery types for training and validation, comprising 81,632 training images and 8240 validation images, while the remaining 72 unseen forgery types form a 243,711-image test set. We follow the released splits and evaluation protocol without additional label supervision.

Each sample is described by structured JavaScript Object Notation (JSON) annotations containing the image path, binary label, forensic subdomain, manipulation type, source dataset, and related metadata. Only the real/fake label is used for optimization; the remaining fields support filtering, statistical analysis, and domain- or forgery-type-level evaluation. Before training and evaluation, we validate annotation fields, labels, image paths, and file readability and exclude invalid or unreadable samples.

All images are converted to red–green–blue (RGB) format and resized to 224×224. Random horizontal flipping is applied during training, whereas validation and testing use deterministic preprocessing. Images are tensorized and normalized channel-wise using mean (0.485,0.456,0.406) and standard deviation (0.229,0.224,0.225).

We follow the released OpenMMSec splits and the unified benchmark implementation provided by ForensicHub [3]. The benchmark was used under its stated research-use terms; availability, licensing, and third-party data conditions are summarized in the Data Availability Statement.

### 3.2. Feature Extraction and Task Adaptation Module

We use DINOv2-Large (ViT-L/14) as the frozen visual backbone and introduce LoRA adapters for task adaptation. Only the adapters and classification head are optimized, enabling parameter-efficient adaptation while leaving the pretrained backbone weights unchanged.

#### 3.2.1. DINOv2 Visual Backbone

We adopt DINOv2 because self-supervised pretraining provides transferable semantic and local representations without task-specific forgery priors. Given an image of size H×W, DINOv2 partitions it into non-overlapping s×s patches. For the 224×224 input resolution and ViT-L/14 backbone used here, s=14 and N=16×16=256. More generally, the number of patch tokens is defined in Equation (1):

(1) $$N=\frac{H}{s}\times \frac{W}{s}.$$

The patch tokens are combined with a pretrained CLS token and positional embeddings and then processed by Transformer encoder blocks. Self-attention and feed-forward networks model token dependencies to produce deep visual representations.

#### 3.2.2. LoRA-Based Parameter-Efficient Adaptation

FID requires task-specific adaptation without unrestricted modification of the foundation model. Training only the classification head leaves the backbone fixed, whereas full-parameter fine-tuning introduces many more trainable degrees of freedom. We therefore use LoRA as an intermediate, restricted adaptation mechanism.

Within SMICA, the pretrained DINOv2 weights remain frozen while low-rank updates are introduced into selected Transformer projections. LoRA therefore controls how task-specific adaptation is performed; the multi-granularity module determines which token-level evidence is aggregated, and ASAM regularizes the resulting trainable parameters.

For an adapted linear mapping, let its pretrained weight matrix be $W_{0}\in R^{d_{\mathrm{in}}\times d_{\mathrm{out}}}$. LoRA freezes $W_{0}$ and introduces two trainable low-rank matrices $A\in R^{d_{\mathrm{in}}\times r}$ and $B\in R^{r\times d_{\mathrm{out}}}$, where $r\ll \min(d_{\mathrm{in}},d_{\mathrm{out}})$. The task-specific update is ΔW=AB. Using row-vector notation, the adapted layer is formulated in Equation (2):

(2) $$y=xW_{0}+\frac{\alpha }{r}xAB+b,$$

where *x* is the input feature, *b* is the bias, *r* is the LoRA rank, and α/r controls update scaling.

LoRA adapters are inserted into the query–key–value (QKV) projection, attention output projection, and the two feed-forward linear layers FC1 and FC2 in each Transformer block. The attention projections govern token-to-token interactions, whereas the feed-forward projections perform channel-wise feature transformation. Updating these mappings with low-rank adapters therefore adapts both interaction and feature transformation while leaving all other pretrained DINOv2 parameters unchanged.

This design is consistent with prior parameter-efficient DINOv2 adaptations that insert low-rank updates into attention projections while retaining the pretrained backbone [38,39]. DINOv2-based forgery detection has likewise applied LoRA-style adapters across Transformer blocks [4]. We additionally adapt the attention output and feed-forward projections so that both attention mixing and channel transformation can undergo restricted task-specific updates.

We keep this functionally motivated insertion configuration fixed across all LoRA-enabled experiments to provide a consistent adaptation architecture when evaluating low-rank adaptation capacity, multi-granularity feature fusion, and ASAM-based optimization. Alternative configurations, such as attention-only, feed-forward-only, or layer-selective adaptation, represent an additional architectural design dimension and were therefore not varied in the present experiments. The selected configuration should thus be understood as a motivated design choice rather than a universally optimal insertion strategy.

After DINOv2-LoRA encoding, the token sequence is represented by Equation (3):

(3) $$T=\left[t_{\mathrm{cls}};p_{1};p_{2};\ldots ;p_{N}\right]\in R^{(N+1)\times C},$$

where $t_{\mathrm{cls}}\in R^{C}$ is the CLS token, $p_{i}\in R^{C}$ denotes the *i*-th patch token, *N* is the number of patch tokens, and *C* is the token dimension. The CLS token captures image-level semantics, while patch tokens retain local cues for subsequent fusion.

### 3.3. Feature Fusion Module

The Feature Fusion Module aggregates complementary token-level evidence from the DINOv2-LoRA output. Because a single token may miss small or sparse artifacts, we combine the CLS token, Patch Mean, and Patch Max to capture global semantics, distributed local statistics, and high-response anomalous cues, respectively. Their concatenation forms a compact multi-granularity representation for detection.

The patch-token matrix extracted from Equation (3) is defined in Equation (4):

(4) $$P=\left[p_{1};p_{2};\ldots ;p_{N}\right]\in R^{N\times C},$$

where *N* is the number of patch tokens and *C* is their dimensionality. Based on the patch-token matrix in Equation (4), Patch Mean summarizes local feature distributions in Equation (5):

(5) $$p_{\mathrm{mean}}=\frac{1}{N}\sum_{j=1}^{N}p_{j}.$$

To retain high-response forensic cues, we perform channel-wise max pooling over the patch tokens, as formulated in Equation (6):

(6) $${\left[p_{\max}\right]}_{c}=\max_{1\leq j\leq N}{\left[p_{j}\right]}_{c},\;c=1,\ldots ,C,$$

where the maximum is computed channel-wise. The three features are concatenated as $T_{\mathrm{fuse}}\in R^{3C}$. The CLS token encodes global semantics, Patch Mean summarizes local statistics, and Patch Max highlights sparse high-response cues. Their combination preserves complementary evidence across representation granularities.

### 3.4. Detection Module

The Detection Module uses a lightweight multilayer perceptron (MLP) to map the fused representation to a binary real/fake prediction. This provides sufficient classification capacity with limited additional parameters. Given $T_{\mathrm{fuse}}^{\left(i\right)}\in R^{3C}$, the head applies Layer Normalization, a linear projection, Gaussian Error Linear Unit (GELU), Dropout, and a final logit projection, as formulated in Equations (7)–(9):

(7) $$\begin{array}{cc} u^{\left(i\right)} & =\mathrm{LN}\left(T_{\mathrm{fuse}}^{\left(i\right)}\right), \end{array}$$

(8) $$\begin{array}{cc} h^{\left(i\right)} & =\mathrm{Dropout}\left(\mathrm{GELU}\left(u^{\left(i\right)}W_{1}+b_{1}\right)\right), \end{array}$$

(9) $$\begin{array}{cc} o_{i} & =h^{\left(i\right)}W_{2}+b_{2}. \end{array}$$

Here, $W_{1}\in R^{3C\times d_{h}}$, $b_{1}\in R^{d_{h}}$, $W_{2}\in R^{d_{h}\times 1}$, $b_{2}\in R$, and $d_{h}$ is the hidden dimension. In our implementation, $d_{h}=512$ and the dropout rate is 0.2.

During inference, ${\hat{p}}_{i}=\sigma \left(o_{i}\right)$ denotes the predicted fake probability. The sample is classified as fake when ${\hat{p}}_{i}\geq \tau$ and as real otherwise. We use a fixed decision threshold of τ=0.5 for ACC and F1 evaluation, with the fake class treated as the positive class.

During training, we use binary focal loss to reduce the contribution of easy samples under imbalanced forensic data. For a mini-batch B, let ${\hat{p}}_{i}$ denote the predicted fake probability and $y_{i}\in \left\{0,1\right\}$ the ground-truth label, where $y_{i}=1$ indicates a fake image. The probability assigned to the ground-truth class and its class-balancing coefficient are defined as

$$p_{i}^{t}=y_{i}{\hat{p}}_{i}+\left(1-y_{i}\right)\left(1-{\hat{p}}_{i}\right),\;\alpha_{i}^{t}=y_{i}\alpha_{f}+\left(1-y_{i}\right)\left(1-\alpha_{f}\right).$$

The focal loss is then formulated as

(10) $$L_{\mathrm{cls}}=-\frac{1}{\left|B\right|}\sum_{i\in B}\alpha_{i}^{t}{\left(1-p_{i}^{t}\right)}^{\gamma }\log\left(p_{i}^{t}\right),$$

where γ is the focusing parameter and $\alpha_{f}$ controls the relative weight assigned to fake samples. The modulating factor down-weights easy examples, while the balancing coefficient adjusts the relative contribution of the two authenticity classes.

### 3.5. Optimization Strategy Module

The DINOv2 backbone $\Theta_{\mathrm{DINO}}$ remains frozen, while the LoRA adapters $\Theta_{\mathrm{LoRA}}$ and classification head $\Theta_{\mathrm{Head}}$ are optimized.

Heterogeneous artifact distributions may cause empirical risk minimization to converge to sharp, distribution-sensitive regions that generalize poorly to unseen forgery types or datasets. We therefore adopt ASAM, which minimizes the worst-case local loss and favors flatter solutions. Let $\theta =\{\Theta_{\mathrm{LoRA}},\Theta_{\mathrm{Head}}\}$ denote the trainable parameters. The ASAM objective is formulated in Equation (11):

(11) $$\min_{\theta }\max_{\epsilon \in S_{\theta }}L\left(\theta +\epsilon \right),$$

where ϵ is a local perturbation and $S_{\theta }$ is scale-adaptive.

ASAM uses adaptive parameter perturbations to account for scale differences between the low-rank adaptation matrices and classification head. It is used only during training, leaving the inference architecture and cost unchanged.

### 3.6. Experimental Setup

#### 3.6.1. Implementation Details

All experiments were conducted on four NVIDIA A40 graphics processing units (GPUs) using PyTorch 2.4.1 and CUDA 12.2. Models were trained for at most 15 epochs with a global batch size of 256 using binary focal loss. The focal-loss parameters were set to γ=2 and $\alpha_{f}=0.5$. AdamW was used with an initial learning rate of $1\times 10^{-4}$, weight decay of $1\times 10^{-4}$, and $\left(\beta_{1},\beta_{2}\right)=\left(0.9,0.999\right)$. Gradient norms were clipped to 1.0. To account for run-to-run variability, all experiments conducted under our implementation were repeated independently using three random seeds: 42, 1024, and 2026. Unless otherwise stated, all reported results produced under our experimental pipeline are the arithmetic mean over these three runs. The same dataset splits, model configurations, and hyperparameters were used across the three runs, with the random seed being the only changed experimental factor. Data loading used eight workers with pinned memory. Early stopping and checkpoint selection were guided by validation AUC.

For SMICA, DINOv2-Large (ViT-L/14) was used as the frozen backbone. LoRA was configured with rank r=16 and scaling factor α=16 unless otherwise stated. The classification head used a hidden dimension of 512 and a dropout rate of 0.2. For the final SMICA configuration, ASAM used a perturbation radius of ρ=0.2, selected from the controlled sensitivity analysis reported in Section 4.4. To disentangle the effects of low-rank adaptation capacity and sharpness-aware optimization, the LoRA and ASAM sensitivity studies were conducted under controlled but distinct settings. For the LoRA rank and scaling-ratio study, ASAM was disabled (ρ=0) for all configurations, while all other training settings were kept unchanged. This setting isolates the effect of the LoRA rank *r* and scaling ratio α/r without the additional influence of sharpness-aware optimization. Conversely, for the ASAM perturbation-radius study, the LoRA configuration was fixed at r=16 and α=16, while only ρ was varied.

A fixed threshold of τ=0.5 was used to convert predicted probabilities into binary labels for ACC and F1; AUC and AP were computed directly from continuous prediction scores.

#### 3.6.2. Baselines and Evaluation Metrics

We compare SMICA with 13 published representative baselines and one full-parameter fine-tuning baseline across the four forensic domains: (1) general visual backbones: SegFormer [40], Swin Transformer [41], and ConvNeXt [42]; (2) Deepfake: Recce [43], SIA [44], and DFF-Adapter [4]; (3) IMD: TruFor [45] and Mesorch [5]; (4) AIGC: Effort [6] and CO-SPY [7]; (5) Doc: FFDN [46]; and (6) pretrained foundation models: iBOT [35] and CLIP [34]. The published baseline values in Table 1 follow the released OpenMMSec benchmark results under the same category taxonomy and averaging protocol. We additionally include two DINOv2-based internal baselines. DINO-Frozen keeps the DINOv2 backbone fully frozen, does not use LoRA or ASAM, and uses CLS + Patch Mean aggregation. DINO-FFT is a full-parameter fine-tuning baseline implemented under our experimental pipeline.

We evaluate Accuracy (ACC), Area Under the Receiver Operating Characteristic (ROC) Curve (AUC), Average Precision (AP), and F1 score, treating the fake class as positive. ACC and F1 use a fixed threshold of τ=0.5. Metrics are computed for each forgery type, averaged within each domain, and then macro-averaged across the four domains for the primary overall evaluation.

To assess robustness to common degradation and post-processing, we evaluate perturbed versions of the test set without retraining or adaptation. We consider four perturbation groups at mild, medium, and strong severity. JPEG compression uses quality factors of 90, 70, and 50; resizing uses scale factors of 0.75, 0.50, and 0.25; and additive Gaussian noise uses standard deviations of 5/255, 10/255, and 20/255. We also define three social-media-like resize–compression proxies: 0.85 + JPEG Q90, 0.70 + JPEG Q80, and 0.50 + JPEG Q70. All robustness results follow the same domain-level and macro-average AUC protocol as the clean test set.

## 4. Results

### 4.1. Comparison and Generalization Study

Table 1 reports category-level, domain-level, and overall AUC on the OpenMMSec test set. Results produced by our experimental pipeline are averaged over three independent runs with random seeds 42, 1024, and 2026. The final SMICA configuration obtains macro-average AUCs of 0.921645, 0.920337, and 0.921344, yielding a mean of 0.9211 and a sample standard deviation of 0.0007 (0.9211±0.0007). The corresponding two-sided 95% confidence interval for the mean, estimated with Student’s *t* distribution, is [0.9194,0.9228], indicating low variation across the evaluated seeds.

SMICA achieves the highest macro-average AUC at 92.1%, compared with 86.4% for DFF-Adapter and 86.2% for DINO-FFT. Among the published baselines, DFF-Adapter has the strongest overall result and achieves 92.6% and 95.0% on Deepfake and AIGC, respectively; SMICA increases these domain-level AUCs to 96.0% and 96.8%.

**Figure 2:**
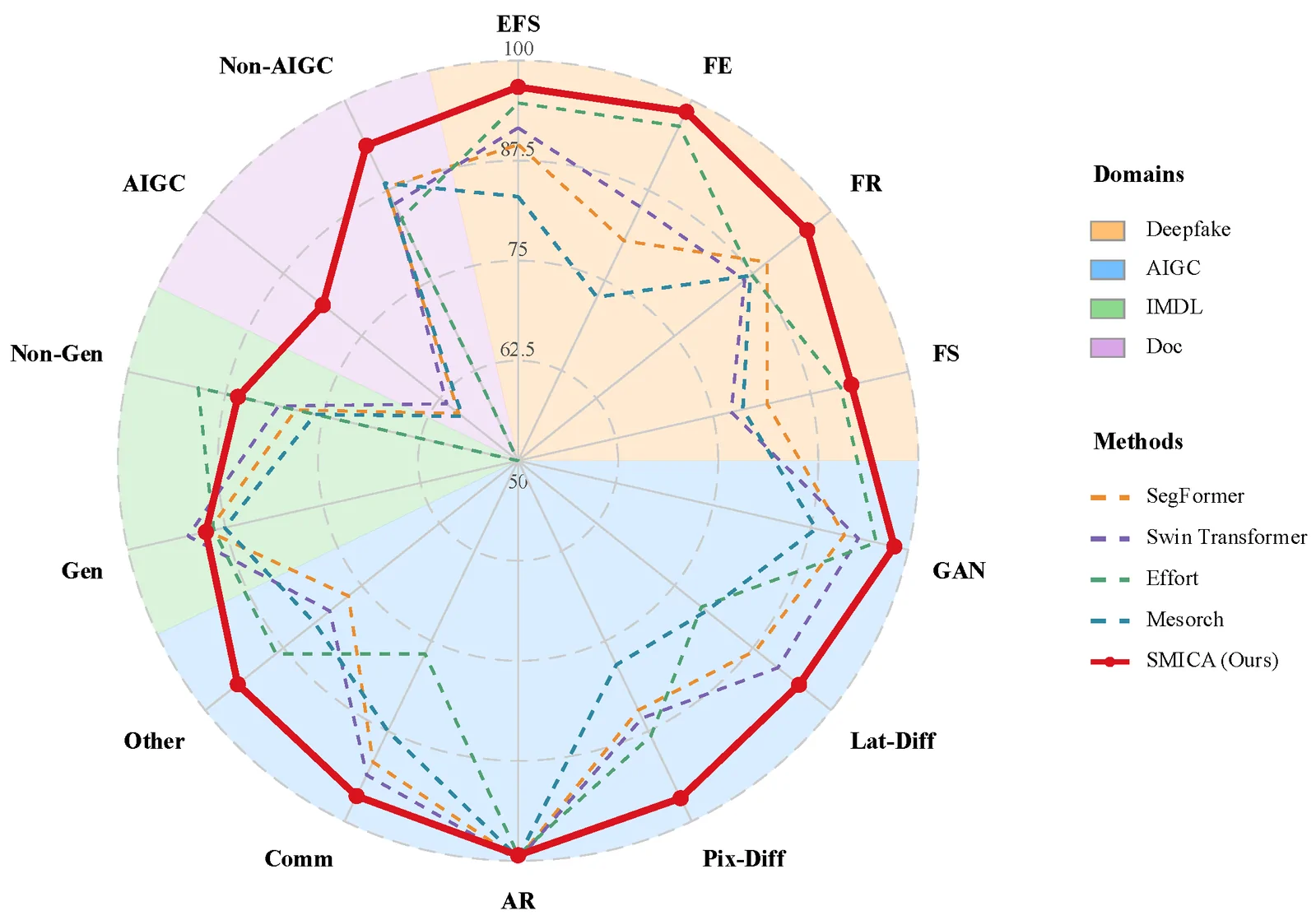
Subdomain-level AUC comparison between SMICA and representative baselines.

At the domain level, SMICA achieves the best AUCs on Deepfake (96.0%), AIGC (96.8%), and Doc (87.4%). On IMD, it reaches 88.0%, below Effort (90.0%) but above FFDN (86.8%). Thus, SMICA provides the strongest overall cross-domain performance while remaining competitive across all four domains. Figure 2 further summarizes the subdomain-level comparison.

### 4.2. Ablation Study

We conduct ablation studies on cross-domain training composition, the contributions of the main framework components, and the internal design of the multi-granularity token aggregation strategy.

#### 4.2.1. Domain Ablation Study

**Figure 3:**
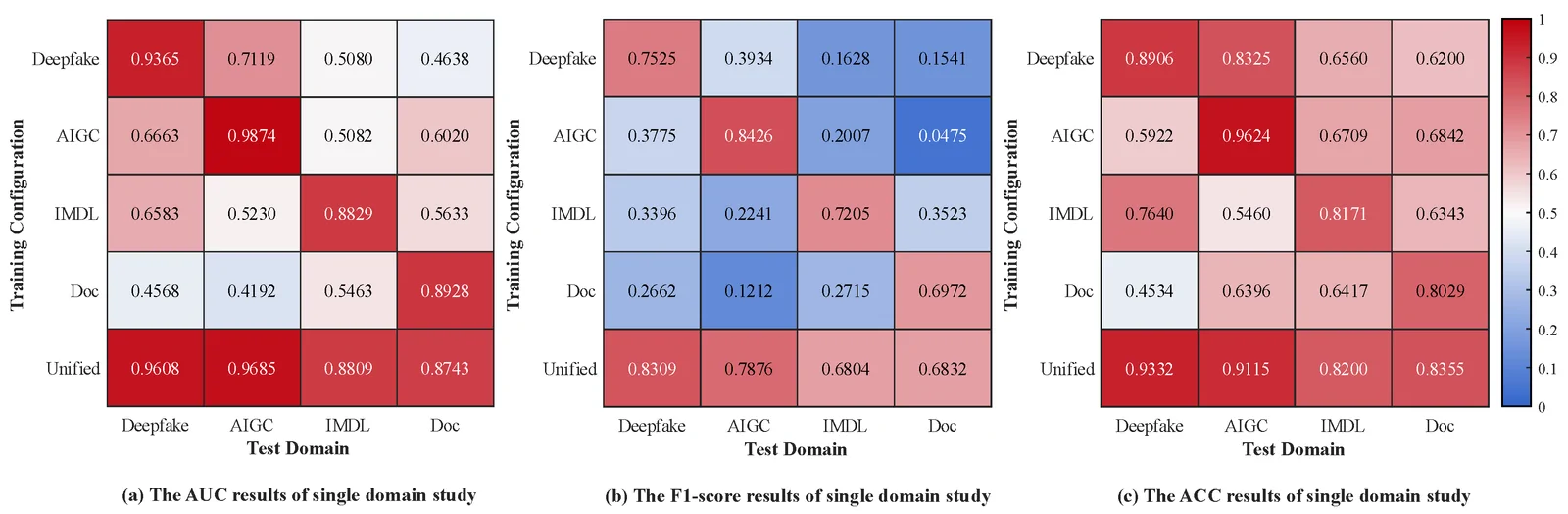
Performance under the Single-Domain (SD) protocol: (**a**) Area Under the Receiver Operating Characteristic Curve (AUC), (**b**) F1, and (**c**) Accuracy (ACC) across the four forensic domains.

We evaluate cross-domain transferability under two protocols: Single-Domain (SD), in which a model is trained on one domain and evaluated on all four, and Leave-One-Domain-Out (LODO), in which a model is trained on three domains and evaluated on the excluded domain. Figure 3 summarizes the SD results.

SD models perform well on their training domain but transfer poorly to others. For example, the Deepfake-trained model attains an AUC of 0.9365 on Deepfake but only 0.5080 on IMD and 0.4638 on Doc. By contrast, the unified model achieves AUCs (F1 scores) of 0.9608 (0.8309), 0.9685 (0.7876), 0.8809 (0.6804), and 0.8743 (0.6832) on Deepfake, AIGC, IMD, and Doc, respectively, indicating substantially more balanced performance.

**Figure 4:**
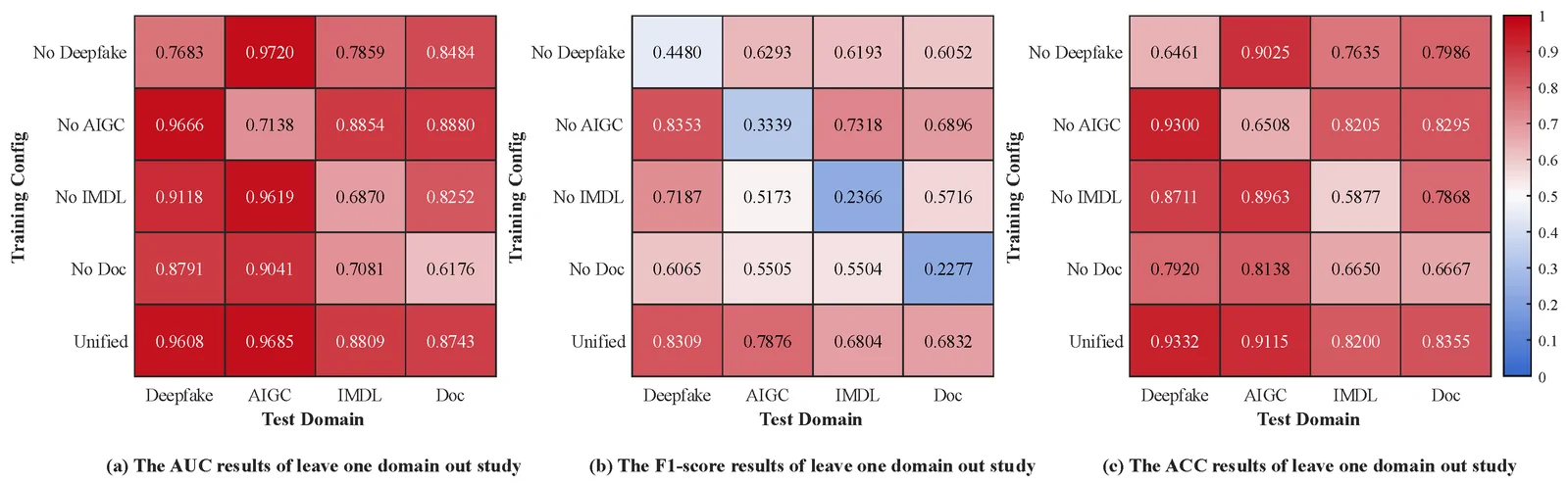
Performance under the Leave-One-Domain-Out (LODO) protocol: (**a**) Area Under the Receiver Operating Characteristic Curve (AUC), (**b**) F1, and (**c**) Accuracy (ACC) when each target domain is excluded from training.

The LODO results are shown in Figure 4.

Excluding the target domain substantially degrades performance. F1 falls to 0.2366 on IMD and 0.2277 on Doc, highlighting the domain specificity of the underlying forensic cues. Nevertheless, LODO generally transfers better than SD training, suggesting that diversity among source domains encourages more transferable representations.

#### 4.2.2. Component-Wise Ablation Study

To isolate the effects of restricted adaptation, multi-granularity aggregation, and sharpness-aware optimization, we conduct a component-wise ablation under the same DINOv2 backbone and unified training protocol. The baseline uses a frozen DINOv2 backbone, CLS + Patch Mean aggregation, and the same MLP head. LoRA, fusion, and ASAM are then enabled individually and in combination. Enabling fusion adds Patch Max, yielding the complete CLS + Patch Mean + Patch Max representation.

Table 2 separates the effects of the three main components. The frozen baseline achieves an AUC of 81.37%. Adding LoRA alone raises AUC to 88.37%, a 7.00 percentage-point gain and the largest individual improvement, indicating that task-specific adaptation is the primary contributor in the unified FID setting.

Adding fusion to the frozen baseline increases AUC from 81.37% to 81.76%, while ASAM alone raises it to 82.34%. These limited gains without LoRA suggest that evidence aggregation and optimization regularization cannot fully compensate for a frozen representation that is mismatched to heterogeneous forensic data. Their benefits become clearer after adaptation: fusion increases the LoRA result from 88.37% to 89.63%, and LoRA + ASAM reaches 91.28%.

The complete configuration achieves the highest AUC of 92.11%. The ablation therefore supports complementary roles for the three components: LoRA adapts the pretrained representation, multi-granularity fusion exposes complementary global and local evidence, and ASAM regularizes the trainable parameters.

#### 4.2.3. Token Aggregation Strategy Ablation Study

We further analyze the fusion design by comparing combinations of the CLS token, Patch Mean, and Patch Max in Table 3.

The isolated CLS token yields an ACC of 0.8664 and AUC of 0.9096, while Patch Mean reaches 0.8696 and 0.9119, respectively, showing the value of distributed patch statistics. Patch Max alone performs worse (ACC: 0.8434, AUC: 0.9038), suggesting that peak responses are more sensitive to localized noise when used in isolation. Its contribution becomes useful when combined with global context and mean patch statistics, where it provides complementary information about sparse high-response regions.

### 4.3. Adaptation Strategy and Feature Space Analysis

**Figure 5:**
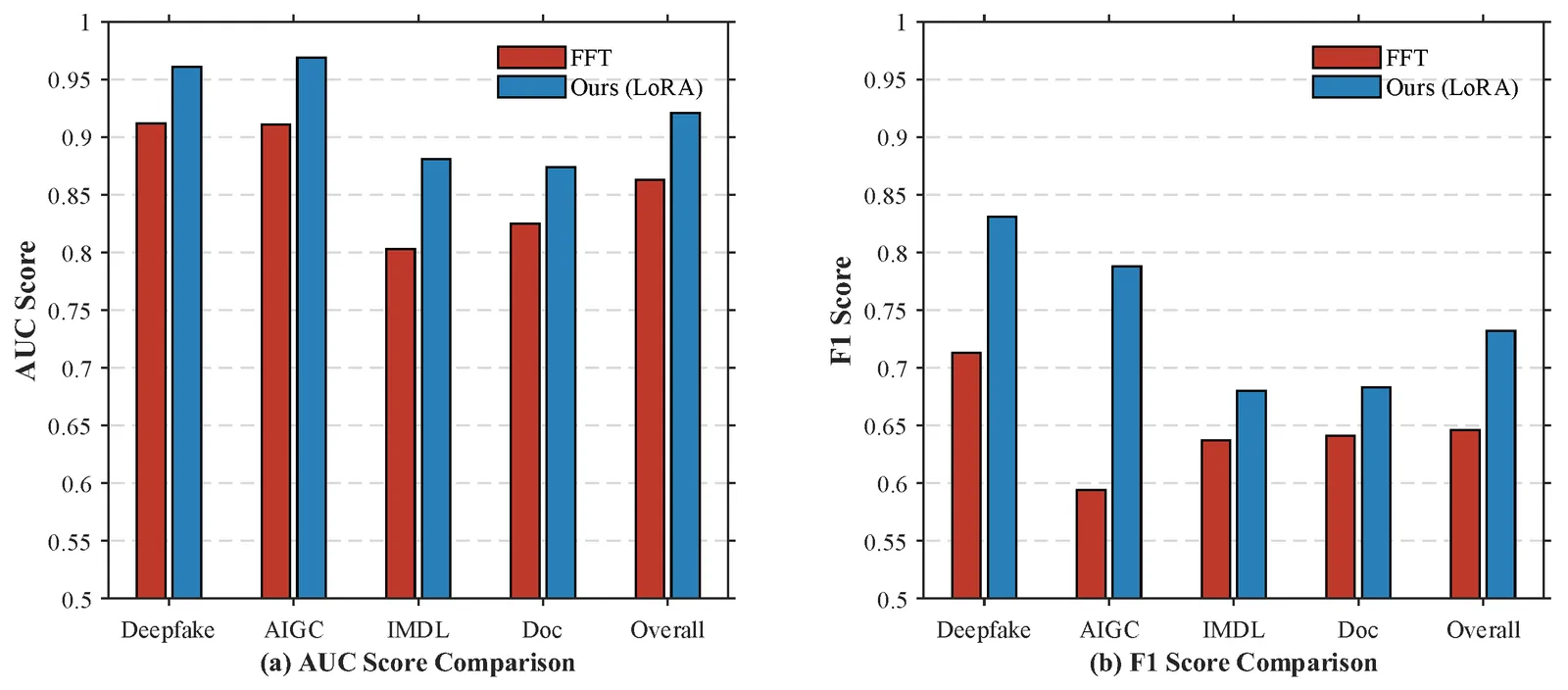
Area Under the Receiver Operating Characteristic Curve (AUC) (**a**) and F1 comparison between full-parameter fine-tuning (DINO-FFT) and SMICA (**b**).

To assess parameter-efficient adaptation, we compare SMICA with full-parameter fine-tuning (DINO-FFT). Figure 5 reports AUC and F1 across all four domains and the overall average.

SMICA achieves higher AUC and F1 across all four domains and in the overall average. Under the present unified FID protocol, restricted low-rank adaptation therefore provides a more effective task-specific update than unrestricted full-parameter fine-tuning.

Table 4 compares the parameter and computational costs of SMICA and DINO-FFT. Both use the same DINOv2-Large backbone family, but SMICA updates only the LoRA adapters and classification head, whereas DINO-FFT optimizes the entire backbone. SMICA contains 314.60M total parameters, of which 7.87M (2.50%) are trainable, compared with 308.31M trainable parameters for DINO-FFT.

Restricted adaptation reduces training cost from 461 G to 183 G FLOPs per image, training time from 93.31 to 38.14 min per epoch, and peak GPU memory from 27.8 to 2.90 GiB. Inference latency is essentially unchanged (23.7 vs. 23.8 ms per image), consistent with LoRA primarily affecting training-time parameterization rather than adding a substantial inference path.

We quantify feature-space structure using the original high-dimensional embeddings immediately before the classification head rather than the two-dimensional t-SNE projections. We report inter-class distance $D_{\mathrm{inter}}$, intra-class distance $D_{\mathrm{intra}}$, intra-class variance $V_{\mathrm{intra}}$, Silhouette coefficient (SIL), and Adjusted Rand Index (ARI). Larger $D_{\mathrm{inter}}$ indicates greater real/fake separation; lower $D_{\mathrm{intra}}$ and $V_{\mathrm{intra}}$ indicate more compact classes; and higher SIL and ARI indicate stronger class-consistent structure.

As shown in Table 5, DINO-Frozen has the weakest separability, with $D_{\mathrm{inter}}=1.1678$, SIL =0.0617, and ARI =0.2276. DINO-FFT improves these metrics to 1.2129, 0.1246, and 0.3541. SMICA further increases $D_{\mathrm{inter}}$ to 1.3105, while reducing $D_{\mathrm{intra}}$ from 0.4744 to 0.2150 and $V_{\mathrm{intra}}$ from 0.3186 to 0.0840 relative to DINO-FFT. It also achieves the highest SIL (0.5802) and ARI (0.4487), indicating stronger real/fake separation and more compact within-class structure.

**Figure 6:**
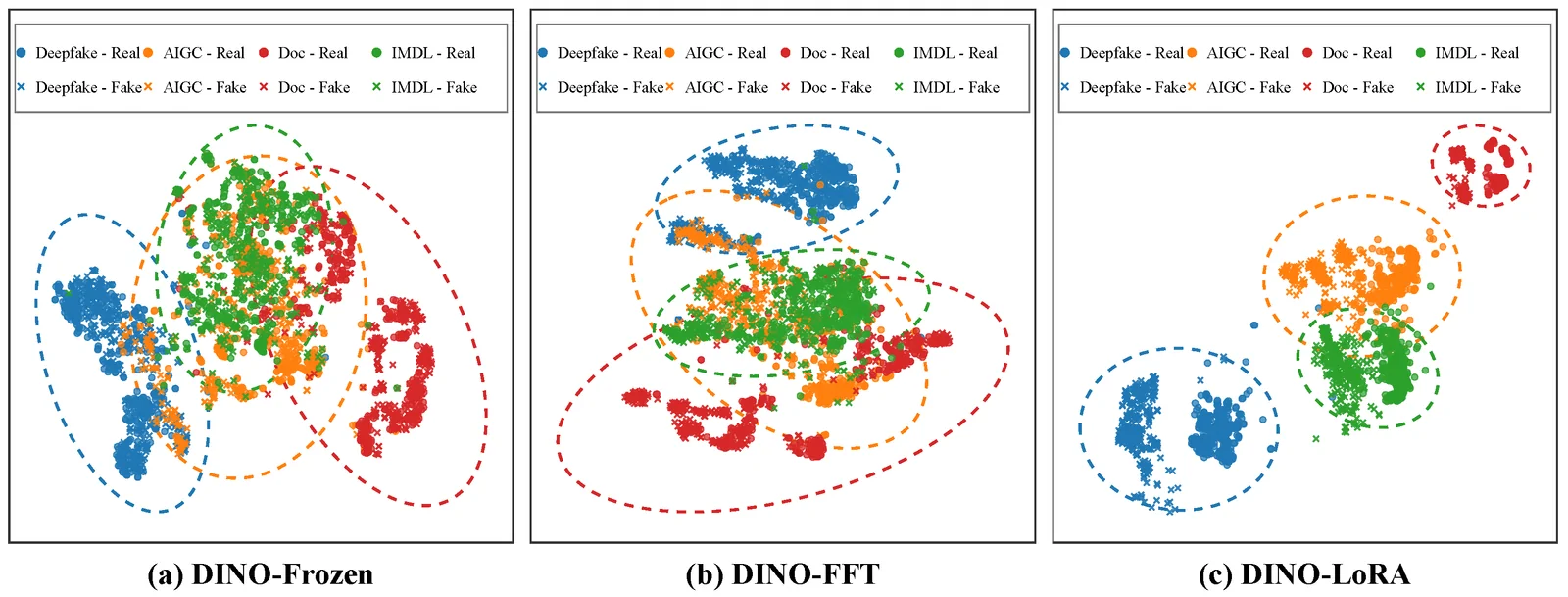
t-Distributed stochastic neighbor embedding (t-SNE) projections of the pre-classification embeddings for DINO-Frozen (**a**), DINO-FFT (**b**), and SMICA (**c**).

Figure 6 visualizes the same pre-classification embeddings with t-SNE. DINO-Frozen shows substantial overlap between real and forged samples, DINO-FFT provides moderate improvement, and SMICA yields a visually clearer distinction between the two authenticity classes, consistent with Table 5.

The t-SNE plots are used only as qualitative supporting evidence. Because t-SNE is nonlinear, hyperparameter-dependent, and does not preserve global distances, we do not infer quantitative inter-class separation or intra-class compactness from the two-dimensional projections; those properties are evaluated in the original feature space in Table 5.

### 4.4. Parameter Sensitivity Study

**Figure 7:**
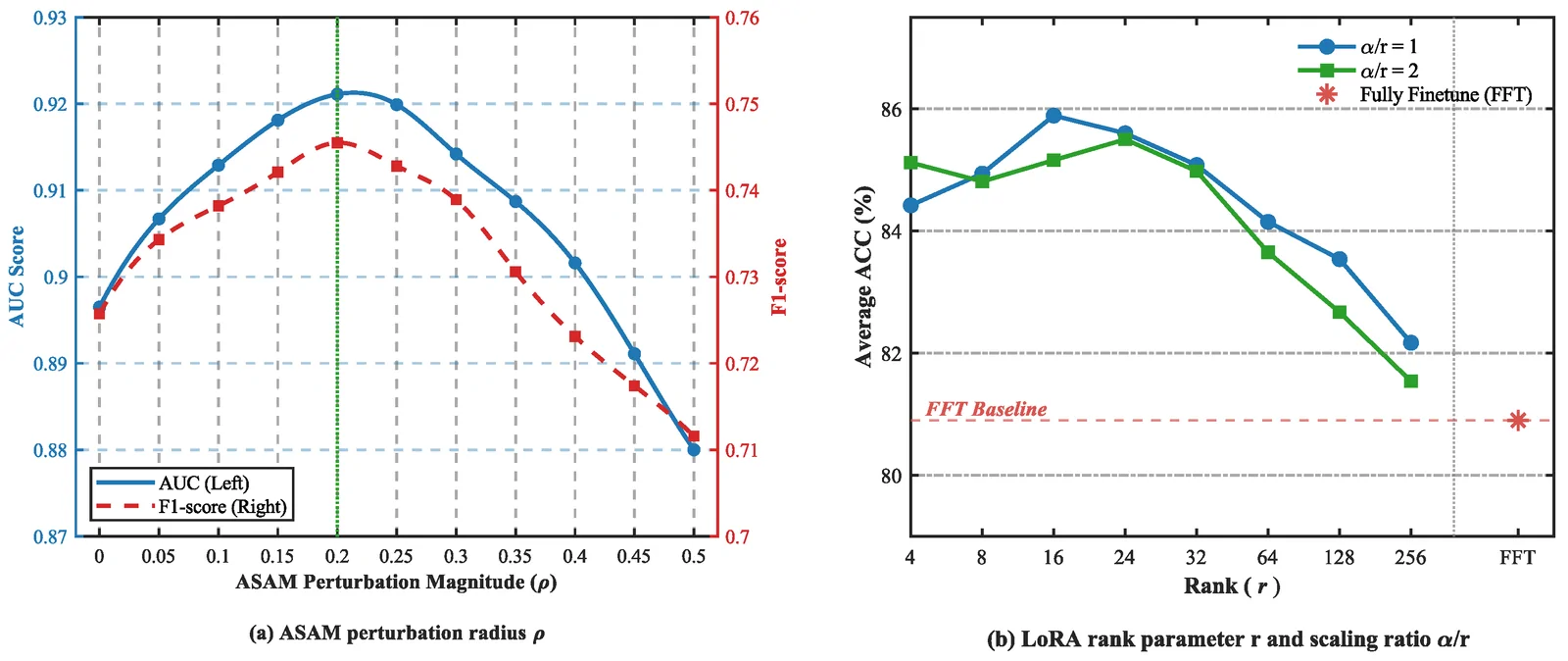
The Adaptive Sharpness-Aware Minimization (ASAM) perturbation radius (**a**) and the controlled sensitivity analysis of the Low-Rank Adaptation (LoRA) rank and scaling ratio (**b**).

Figure 7 summarizes the sensitivity to LoRA rank *r*, scaling ratio α/r, and ASAM perturbation radius ρ.

To isolate LoRA capacity from sharpness-aware optimization, the rank and scaling-ratio study disables ASAM (ρ=0) while keeping all other model and training settings fixed. Only *r* and α/r are varied. A small rank limits adaptation capacity, whereas an excessively large rank introduces additional degrees of freedom that may increase overfitting to seen forgeries.

Without ASAM, performance peaks at α/r=1 and r=16, with an average ACC of 85.89% across the three seeds, 4.99 percentage points above full-parameter fine-tuning (80.90%). Increasing the rank further provides no benefit, and ACC falls to 82.17% at r=256. We therefore use r=16 and α=16 by default.

The 85.89% ACC above corresponds to the no-ASAM sensitivity setting and is not the final SMICA result. With ASAM enabled at ρ=0.2, the complete model achieves 87.51% ACC, as reported in Table 3.

For the ASAM study, LoRA is fixed at r=16 and α=16, and only ρ is varied. Table 6 reports overall and domain-level results. At ρ=0, the model reduces to the corresponding non-sharpness-aware baseline and obtains an AUC of 0.8963. Performance improves up to ρ=0.20, where the model reaches the highest macro-average AUC (0.9211) and F1 (0.7455), together with the best domain-level results among the evaluated radii.

Performance gradually declines beyond ρ=0.20, indicating that moderate sharpness-aware perturbation is beneficial whereas larger perturbations can move optimization outside a useful local neighborhood. Relative to ρ=0, the selected ρ=0.2 improves AUC by 0.0248 and F1 by 0.0198, showing an additional benefit beyond the fixed LoRA configuration.

The LoRA rank *r* and ASAM perturbation radius ρ may also interact during optimization. The LoRA rank determines the capacity of the trainable low-rank updates, whereas the ASAM perturbation radius controls the extent of scale-adaptive parameter perturbations. Changing the LoRA rank alters the dimensionality and parameterization of the adaptation space, potentially affecting the local loss landscape and parameter sensitivity. Consequently, the same perturbation radius may impose different effective optimization constraints at different ranks, and the appropriate perturbation strength may vary with adaptation capacity without necessarily following a monotonic relationship. The present controlled analyses characterize the sensitivity of LoRA rank with ASAM disabled (ρ=0) and the sensitivity of ASAM perturbation radius at a fixed rank (r=16), providing empirical support for the selected configuration without establishing its joint optimality.

### 4.5. Robustness to Image Degradation and Post-Processing

We further evaluate SMICA under degradations and post-processing operations that may occur during image transmission and redistribution. Perturbations are applied only at test time; the trained model and inference settings remain unchanged. Table 7 reports domain-level and macro-average AUC under JPEG compression, resizing, Gaussian noise, and composite social-media-like processing at three severity levels.

Performance decreases consistently as perturbation severity increases. JPEG compression causes the smallest degradation: at Q90, SMICA retains a macro-average AUC of 91.10%, only 1.01 percentage points below the clean result. The score decreases to 88.75% at Q70 and 86.29% at Q50, indicating progressive loss of discriminative forensic information under stronger recompression.

Resizing and Gaussian noise cause larger degradation at high severity. A scale factor of 0.25 reduces macro-average AUC to 82.76%, while Gaussian noise with σ=20/255 yields 84.84%. Sensitivity also differs by domain: IMD falls from 88.09% on clean images to 71.17% under the strongest resizing condition, consistent with greater vulnerability of localized manipulation cues to severe spatial information loss.

The composite social-media proxy produces the largest overall degradation: macro-average AUC decreases from 87.25% under the mild setting (Resize ×0.85 + JPEG Q90) to 84.71% under the medium setting and 80.52% under the strongest setting (Resize ×0.50 + JPEG Q70). SMICA is therefore relatively stable under mild perturbations, especially mild JPEG compression, but remains sensitive to severe spatial degradation and compound post-processing.

### 4.6. Case Study

**Figure 8:**
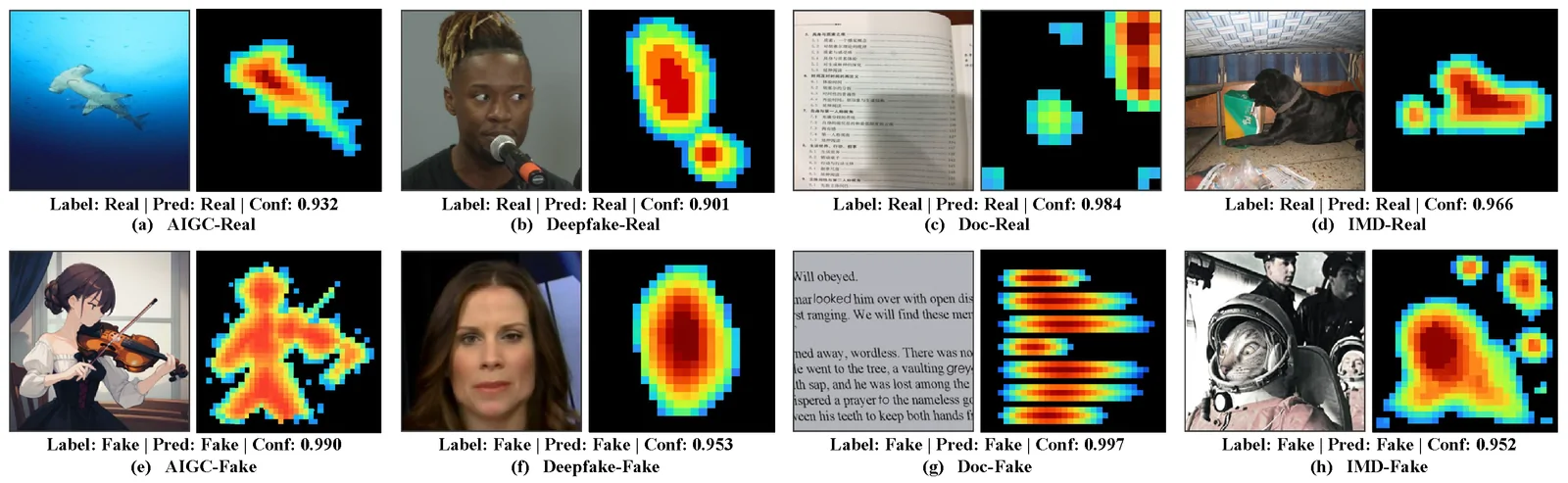
Representative Grad-CAM visualizations from the four Fake Image Detection (FID) domains. AIGC-Real (**a**); Deepfake-Real (**b**); Doc-Real (**c**); IMD-Real (**d**); AIGC-Fake (**e**); Deepfake-Fake (**f**); Doc-Fake (**g**); IMD-Fake (**h**).

Figure 8 presents representative cases from the four FID domains. For each example, Grad-CAM is computed from the final DINOv2 layer to visualize activation patterns associated with the model prediction. These examples are qualitative and are not intended as a quantitative localization or attribution evaluation.

For Deepfake images, high-response regions often appear around facial boundaries and components. AIGC examples frequently emphasize object contours and edge-texture regions, while IMD examples show activations near edited or structurally inconsistent areas. Document examples often emphasize text, stamps, or signatures. These visualizations illustrate response patterns only and are not used as evidence of localization accuracy, causal attribution, or shortcut avoidance.

## 5. Discussion

### 5.1. Interpretation of the Main Results

The results indicate that unified training benefits from representations that preserve transferable visual structure while adapting to task-specific artifacts. SMICA reaches a macro-average AUC of 0.9211 and achieves the strongest evaluated domain-level results on Deepfake, AIGC, and Doc. Its advantage over DINO-FFT is notable because both use the same DINOv2 family, whereas SMICA updates only a small subset of parameters. Under the present benchmark, unrestricted full-parameter adaptation is therefore unnecessary for achieving the best overall result.

Performance remains uneven across domains. SMICA performs strongly on Deepfake, AIGC, and Doc, but its IMD AUC (88.0%) remains below Effort (90.0%). This gap is important because IMD often involves highly localized modifications whose discriminative traces occupy only a small image region.

We do not attribute this behavior to input resolution alone. Sensitivity to localized manipulation evidence may also depend on patch-token granularity, image-level binary supervision, and feature aggregation. Because these factors are not isolated experimentally, the IMD result should be treated as a limitation of the current image-level formulation rather than evidence for a single cause.

SMICA targets unified image-level detection rather than specialized pixel-level localization. The IMD gap therefore marks an important boundary of the current formulation, while the macro-average results show that a single model can remain competitive across all four domains. Specialized IMD methods demonstrate the value of multi-scale, local, and dense forensic information for subtle manipulations [5,18,19]. Recent self-supervised vision foundation models similarly emphasize high-quality dense spatial representations; for example, DINOv3 discusses the balance between high-level global representations and dense features [47]. These observations motivate finer-grained or multi-resolution representations for future unified FID systems.

The token ablation also clarifies the role of multi-granularity fusion. Patch Max alone and its pairwise combinations do not consistently improve performance, suggesting that isolated peak activations are sensitive to local noise or domain-specific responses. Patch Mean provides a more stable summary of distributed local evidence, while the CLS token retains image-level semantics. The best result occurs when all three are combined, indicating that sparse high-response cues are most useful when contextualized by both global information and average local statistics. Likewise, the ASAM study shows that moderate perturbations improve AUC and F1, whereas larger perturbations reduce performance, supporting its role as a regularizer.

### 5.2. Practical and Methodological Significance

A practical advantage of SMICA is that one monolithic detector covers four substantially different fake-image domains without separate expert models or domain-specific routing at inference. The frozen DINOv2 backbone provides a shared feature space, while LoRA confines task-specific changes to low-rank subspaces. As a result, only 7.87 M parameters (2.50% of the total) are trainable. ASAM is used only during training and adds no inference module.

Methodologically, the results support combining evidence across representation granularities. Global semantics capture large-scale inconsistencies, distributed patch statistics summarize local irregularities, and channel-wise maxima retain sparse high-response cues. SMICA does not optimize domain separation; the learning objective remains binary real/fake discrimination, and domain labels are used only for analysis and evaluation. The results therefore favor retaining complementary local information rather than reducing a foundation-model representation to a single global token.

### 5.3. Limitations and Future Work

Several limitations qualify these findings. First, the OpenMMSec test split evaluates 72 unseen forgery types within four established forensic domains; the results therefore demonstrate unseen-forgery-type generalization rather than unrestricted open-world generalization to entirely new domains. Second, the model uses fixed-resolution, image-level preprocessing and supervision. Its weaker performance on highly localized manipulations may reflect input resolution, patch granularity, feature aggregation, and the absence of localization supervision, but these factors are not disentangled here. Third, because training is based on image-level binary labels, the case-study activation maps provide qualitative interpretability rather than a localization guarantee. Fourth, the robustness evaluation uses controlled JPEG compression, resizing, Gaussian noise, and composite resize–compression proxies. Real-world pipelines may introduce more complex shifts through platform-specific processing, repeated transformations, screenshots, and device-dependent acquisition artifacts.

Future work will investigate higher- or multi-resolution representations for small forged regions, joint detection-localization objectives, and more realistic transmission pipelines, including platform-specific processing, repeated compression and resizing, screenshots, and device-dependent acquisition artifacts. We also plan to evaluate newly emerging generators and manipulation mechanisms, broaden statistical validation across additional benchmarks and training conditions, and examine whether patch-level constraints can improve interpretability and localization without sacrificing parameter efficiency.

## 6. Conclusions

We presented SMICA, a unified framework for fake image detection across heterogeneous forensic domains. A frozen DINOv2 backbone with LoRA restricts task-specific adaptation, fusion of the CLS token, Patch Mean, and Patch Max integrates complementary evidence across granularities without additional learnable parameters, and ASAM regularizes the trainable parameters. Under the OpenMMSec protocol, SMICA achieves a macro-average AUC of 0.9211 while optimizing only 2.50% of the total parameters, outperforming the evaluated published baselines and DINO-FFT in overall AUC. Component and token ablations, sensitivity analysis, feature-space metrics, and robustness experiments support the complementary roles of restricted adaptation, multi-granularity aggregation, and optimization regularization. The remaining gap in highly localized manipulations and the degradation under severe post-processing motivate future work on finer-grained representations, localization-aware supervision, and more realistic image-transmission pipelines.

## Figures and Tables

**Table 1:** Category-level, domain-level, and macro-average AUC results (%) for baseline models and SMICA on OpenMMSec.

Method	Deepfake					AIGC							IMD			Doc			Avg
	EFS	FE	FR	FS	Avg	GAN	L-Diff	P-Diff	AR	Comm	Oth	Avg	Gen	N-Gen	Avg	AIGC	N-AIGC	Avg	
SegFormer [40]	89.5	80.5	89.8	81.9	85.4	91.8	88.0	84.6	99.8	91.8	77.1	88.9	89.7	78.5	84.1	59.5	87.9	73.7	83.0
Swin Transformer [41]	91.6	85.2	86.2	77.3	85.1	93.6	91.5	85.7	99.9	93.6	80.1	90.7	92.3	80.7	86.5	61.4	85.6	73.5	84.0
ConvNeXt [42]	84.7	84.4	90.0	80.3	84.9	92.5	87.0	84.3	99.9	92.2	75.0	88.5	89.5	75.3	82.4	59.5	84.3	71.9	81.9
Recce [43]	68.9	54.1	51.0	55.4	57.4	83.2	71.2	70.2	94.2	67.9	74.4	76.9	59.6	55.2	57.4	62.1	66.5	64.3	64.0
SIA [44]	67.4	53.7	81.0	68.1	67.6	86.3	74.4	74.9	96.7	77.7	81.9	82.0	79.3	55.9	67.6	58.8	89.1	74.0	72.8
DFF-Adapter [4]	93.5	94.2	91.9	90.8	92.6	96.8	92.5	95.0	98.2	94.5	92.8	95.0	81.5	75.8	78.6	73.2	85.5	79.3	86.4
TruFor [45]	78.6	65.1	83.1	69.8	74.2	88.0	84.8	77.1	99.2	88.8	81.0	86.5	90.7	78.2	84.5	51.8	90.5	71.2	79.1
Mesorch [5]	83.0	72.7	87.1	78.8	80.4	88.2	80.1	78.3	99.6	87.5	82.5	86.0	87.6	76.0	81.8	58.9	88.5	73.7	80.5
FFDN [46]	76.2	51.5	87.2	76.3	72.8	95.9	92.9	94.9	100.0	93.3	74.8	92.0	93.3	80.2	86.8	56.1	84.7	70.4	80.5
Effort [6]	94.7	96.4	87.3	91.3	92.4	95.9	79.3	88.2	99.3	76.8	88.7	88.0	89.0	91.0	**90.0**	48.3	83.6	66.0	84.1
CO-SPY [7]	80.9	62.3	85.1	71.4	74.9	92.4	88.8	85.5	98.9	94.1	70.2	88.3	84.4	68.0	76.2	52.2	85.7	69.0	77.1
iBOT [35]	82.9	81.3	85.7	73.5	80.9	87.8	81.8	82.3	94.3	86.0	85.2	86.3	77.6	68.7	73.1	67.1	87.0	77.0	79.3
CLIP [34]	81.8	66.9	85.7	74.9	77.3	95.4	90.4	88.8	100.0	93.1	76.8	90.8	87.8	70.8	79.3	56.9	89.2	73.1	80.1
DINO-Frozen	87.2	83.5	81.8	82.7	83.8	90.8	85.5	87.8	88.2	86.5	88.8	87.9	77.5	72.3	74.9	72.5	85.2	78.9	81.3
DINO-FFT	91.2	93.3	92.1	88.2	91.2	93.0	87.7	91.3	95.0	90.8	88.9	91.1	83.0	77.5	80.2	77.1	87.8	82.4	86.2
**SMICA**	96.7	98.4	96.2	92.7	**96.0**	98.2	94.9	96.8	99.3	96.5	94.8	**96.8**	90.0	85.9	88.0	81.2	93.7	**87.4**	**92.1**

**Note:** In the four domain-level Avg columns and the overall Avg column, the best results are highlighted in **bold**, and the second-best results are underlined. DINO-Frozen denotes a fully frozen DINOv2 backbone without LoRA or ASAM and uses CLS + Patch Mean aggregation. EFS: Entire Face Synthesis; FE: Face Editing; FR: Face Reenactment; FS: Face Swap; GAN: Generative Adversarial Network; L-Diff: Latent Diffusion; P-Diff: Pixel-Space Diffusion; AR: Autoregressive; Comm: Commercial; Oth: Other; Gen: Generative; N-Gen: Non-Generative; N-AIGC: Non-AIGC. Category groupings follow the released OpenMMSec taxonomy.

**Table 2:** Component-wise ablation of LoRA, multi-granularity fusion, and ASAM under the unified OpenMMSec evaluation protocol.

Configuration	LoRA	Fusion	ASAM	AUC (%)
Baseline	**✗**	**✗**	**✗**	81.37
Baseline + LoRA	**✔**	**✗**	**✗**	88.37
Baseline + Fusion	**✗**	**✔**	**✗**	81.76
Baseline + ASAM	**✗**	**✗**	**✔**	82.34
Baseline + LoRA + Fusion	**✔**	**✔**	**✗**	89.63
Baseline + LoRA + ASAM	**✔**	**✗**	**✔**	91.28
Baseline + Fusion + ASAM	**✗**	**✔**	**✔**	82.98
**SMICA**	**✔**	**✔**	**✔**	**92.11**

**Note:** The baseline consists of a frozen DINOv2 backbone, CLS + Patch Mean aggregation, and the MLP classification head. “Fusion” denotes the complete multi-granularity aggregation using CLS + Patch Mean + Patch Max. When LoRA is disabled, the DINOv2 backbone remains frozen and ASAM, when enabled, operates on the remaining trainable classification head. All configurations use the same data splits and training protocol. **✔** and **✗** indicate that a component is enabled and disabled, respectively. The best result is highlighted in **bold**, and the second-best result is underlined.

**Table 3:** Ablation results of different token aggregation strategies.

Token Components			ACC	AUC	AP	F1
CLS	Patch Mean	Patch Max				
**✔**	**✗**	**✗**	0.8664	0.9096	0.8095	0.7281
**✗**	**✔**	**✗**	0.8696	0.9119	0.8145	0.7317
**✗**	**✗**	**✔**	0.8434	0.9038	0.7952	0.7042
**✔**	**✗**	**✔**	0.8372	0.8928	0.7756	0.6854
**✗**	**✔**	**✔**	0.8401	0.8760	0.7461	0.6651
**✔**	**✔**	**✗**	0.8703	0.9128	0.8259	0.7298
**✔**	**✔**	**✔**	**0.8751**	**0.9211**	**0.8321**	**0.7455**

**Note:✔** and **✗** indicate that a token component is included and excluded, respectively. The best results are highlighted in **bold**, and the second-best results are underlined.

**Table 4:** Parameter and computational efficiency comparison between SMICA and DINO-FFT under the same experimental environment.

Method	Total Params	Trainable Params	Trainable Ratio	Training FLOPs/Image	Training Time/Epoch ↓	Inference Time/Image ↓	Peak GPU Memory ↓
DINO-FFT	308.31 M	308.31 M	100.00%	461 G	93.31 min	23.8 ms	27.8 GiB
**SMICA**	314.60 M	**7.87 M**	**2.50%**	**183 G**	**38.14 min**	**23.7 ms**	**2.90 GiB**

**Note:** SMICA and DINO-FFT were profiled using the same hardware and software environment, input resolution, batch-size configuration, and numerical precision. Training FLOPs are reported per image, training time is reported per epoch, inference latency is averaged per image, and peak GPU memory is measured during training. ↓ indicates that a lower value is better.

**Table 5:** Quantitative comparison of feature-space structure using the original high-dimensional embeddings before t-SNE projection.

Method	$D_{\mathrm{inter}}↑$	$D_{\mathrm{intra}}↓$	$V_{\mathrm{intra}}↓$	SIL↑	ARI↑
DINO-Frozen	1.1678	0.5325	0.3393	0.0617	0.2276
DINO-FFT	1.2129	0.4744	0.3186	0.1246	0.3541
**SMICA**	**1.3105**	**0.2150**	**0.0840**	**0.5802**	**0.4487**

**Note:** All metrics are computed in the original feature space before t-SNE projection. $D_{\mathrm{inter}}$: inter-class distance; $D_{\mathrm{intra}}$: intra-class distance; $V_{\mathrm{intra}}$: intra-class variance; SIL: Silhouette coefficient; ARI: Adjusted Rand Index. ↑ indicates that a higher value is better, whereas ↓ indicates that a lower value is better. The best results are highlighted in **bold**, and the second-best results are underlined.

**Table 6:** Sensitivity analysis of the ASAM perturbation radius ρ. The overall AUC and F1 are reported together with domain-level AUC (%) on Deepfake, AIGC, IMD, and Doc.

ρ	Overall		Domain-Level AUC (%)			
	AUC	F1	Deepfake	AIGC	IMD	Doc
0.00	0.8963	0.7257	93.46	94.24	85.70	85.12
0.05	0.9065	0.7343	94.52	95.31	86.68	86.09
0.10	0.9127	0.7382	95.17	95.96	87.27	86.68
0.15	0.9179	0.7421	95.71	96.51	87.77	87.17
**0.20**	**0.9211**	**0.7455**	**96.08**	**96.85**	**88.09**	**87.43**
0.25	0.9193	0.7428	95.88	96.68	87.89	87.29
0.30	0.9136	0.7389	95.28	96.08	87.34	86.75
0.35	0.9081	0.7306	94.71	95.50	86.81	86.22
0.40	0.9010	0.7231	93.97	94.75	86.13	85.55

**Note:** The best result in each column is highlighted in **bold**. The LoRA configuration is fixed at r=16 and α=16, while all other training settings remain unchanged.

**Table 7:** Robustness of SMICA under different image degradation and post-processing conditions. Domain-level and macro-average AUC values are reported in percent (%).

Condition	Group	Severity	Deepfake	AIGC	IMD	Doc	Macro AUC	Δ Macro
Clean	Clean	Clean	**96.08**	**96.85**	**88.09**	**87.43**	**92.11**	–
JPEG-Q90	JPEG	Mild	94.92	95.61	86.54	87.35	91.10	−1.01
JPEG-Q70	JPEG	Medium	94.17	92.44	83.24	85.15	88.75	−3.36
JPEG-Q50	JPEG	Strong	92.90	89.89	80.59	81.79	86.29	−5.82
Resize-0.75	Resize	Mild	94.30	94.37	82.11	84.87	88.91	−3.20
Resize-0.50	Resize	Medium	93.09	94.62	80.28	82.22	87.55	−4.56
Resize-0.25	Resize	Strong	88.25	93.84	71.17	77.80	82.76	−9.35
Noise-5/255	Gaussian Noise	Mild	96.27	91.17	84.43	85.63	89.37	−2.74
Noise-10/255	Gaussian Noise	Medium	93.94	88.87	80.43	83.67	86.73	−5.38
Noise-20/255	Gaussian Noise	Strong	90.09	87.19	78.87	83.21	84.84	−7.27
Social-Mild	Social-media Proxy	Mild	92.48	90.61	81.62	84.29	87.25	−4.86
Social-Medium	Social-media Proxy	Medium	91.57	88.57	77.95	80.77	84.71	−7.40
Social-Strong	Social-media Proxy	Strong	89.62	85.23	70.39	76.85	80.52	−11.59

**Note:** All perturbations are applied only at test time without model retraining or adaptation. JPEG compression uses quality factors of 90, 70, and 50 for mild, medium, and strong settings, respectively. Resize factors are 0.75, 0.50, and 0.25. Gaussian noise uses standard deviations of 5/255, 10/255, and 20/255. The social-media proxy combines resizing and JPEG compression: mild = Resize ×0.85 + JPEG Q90; medium = Resize ×0.70 + JPEG Q80; strong = Resize ×0.50 + JPEG Q70. Δ Macro denotes the absolute change in macro-average AUC relative to the clean condition (92.11%).

## Data Availability

The OpenMMSec benchmark used in this study is available on the Tianchi platform at https://tianchi.aliyun.com/dataset/210595 (accessed on 9 September 2026). Because OpenMMSec aggregates multiple third-party forensic datasets, the constituent source data remain subject to the licenses, access conditions, privacy requirements, and redistribution restrictions specified by their original providers. The ForensicHub codebase used to implement the unified benchmark protocol is publicly available at https://github.com/scu-zjz/ForensicHub (accessed on 9 September 2026) under a CC BY 4.0 license.

## Abbreviations

The following abbreviations are used in this manuscript:

ACC	Accuracy
AIGC	AI-Generated Content
AP	Average Precision
ASAM	Adaptive Sharpness-Aware Minimization
AUC	Area Under the Receiver Operating Characteristic Curve
CLS	Classification Token
Doc	Document Image Forgery Detection
FID	Fake Image Detection
FFT	Full-Parameter Fine-Tuning
GAN	Generative Adversarial Network
GELU	Gaussian Error Linear Unit
GPU	Graphics Processing Unit
IMD	Image Manipulation Detection
JSON	JavaScript Object Notation
LODO	Leave-One-Domain-Out
LoRA	Low-Rank Adaptation
MLLM	Multimodal Large Language Model
MLP	Multilayer Perceptron
QKV	Query–Key–Value
RGB	Red–Green–Blue
ROC	Receiver Operating Characteristic
SAM	Segment Anything Model
SD	Single-Domain
ViT	Vision Transformer
